# RETRACTION: “Platelet-Rich Plasma Combined with Alendronate Reduces Pain and Inflammation in Induced Osteoarthritis in Rats by Inhibiting the Nuclear Factor-Kappa B Signaling Pathway”

**DOI:** 10.1155/bmri/9840675

**Published:** 2025-06-10

**Authors:** BioMed Research International

**RETRACTION**: F. Xin, H. Wang, F. Yuan, and Y. Ding, “Platelet-Rich Plasma Combined with Alendronate Reduces Pain and Inflammation in Induced Osteoarthritis in Rats by Inhibiting the Nuclear Factor-Kappa B Signaling Pathway,” *BioMed Research International*, 2020, no. 1 (2020): 1–10, https://doi.org/10.1155/2020/8070295.

The above article, published online on 28 September 2020 in Wiley Online Library (http://wileyonlinelibrary.com), has been retracted following the agreement of the journal's Section Editor, Jinsong Ren; and John Wiley & Sons Ltd.

The retraction has been agreed following an investigation undertaken by the publisher, which identified concerns in Figure 1a of the manuscript. More specifically, unexpected areas of overlap were identified between the Control and OA panels as well as RP and ALN + PRP panels.

The authors were contacted regarding these concerns but did not provide a response. As a result of the investigation, the conclusions of the article are considered unreliable.

